# Factors necessary to produce basoapical polarity in human glandular epithelium formed in conventional and high-throughput three-dimensional culture: example of the breast epithelium

**DOI:** 10.1186/1741-7007-7-77

**Published:** 2009-11-16

**Authors:** Cedric Plachot, Lesley S Chaboub, Hibret A Adissu, Lei Wang, Albert Urazaev, Jennifer Sturgis, Elikplimi K Asem, Sophie A Lelièvre

**Affiliations:** 1 Department of Basic Medical Sciences and Purdue Center for Cancer Research; Purdue University, West Lafayette, IN 47907, USA; 2 Department of Biological Sciences; Purdue University, West Lafayette, IN 47907, USA

## Abstract

**Background:**

Basoapical polarity in epithelia is critical for proper tissue function, and control of proliferation and survival. Cell culture models that recapitulate epithelial tissue architecture are invaluable to unravel developmental and disease mechanisms. Although factors important for the establishment of basal polarity have been identified, requirements for the formation of apical polarity in three-dimensional tissue structures have not been thoroughly investigated.

**Results:**

We demonstrate that the human mammary epithelial cell line-3522 S1, provides a resilient model for studying the formation of basoapical polarity in glandular structures. Testing three-dimensional culture systems that differ in composition and origin of substrata reveals that apical polarity is more sensitive to culture conditions than basal polarity. Using a new high-throughput culture method that produces basoapical polarity in glandular structures without a gel coat, we show that basal polarity-mediated signaling and collagen IV are both necessary for the development of apical polarity.

**Conclusion:**

These results provide new insights into the role of the basement membrane, and especially collagen IV, in the development of the apical pole, a critical element of the architecture of glandular epithelia. Also, the high-throughput culture method developed in this study should open new avenues for high-content screening of agents that act on mammary tissue homeostasis and thus, on architectural changes involved in cancer development.

## Background

Three-dimensional (3D) cell culture is defined as the culture of cells in the presence of an extracellular milieu that promotes the formation of multicellular structures in the x, y and z axis [1]. Three-dimensional culture permits the study of epithelial cell arrangement into tissue structures, and the investigation of pathways critical for the establishment and maintenance of structural and functional aspects of tissue differentiation. However, the basoapical tissue polarity axis, a critical feature of normal epithelial differentiation, remains difficult to replicate with human cell systems.

In glandular and tubular epithelial structures, in which epithelial cells are organized as one layer surrounding a lumen, the establishment of apical polarity characterized by the formation of cell-cell adherens and tight junctions accompanies lumen formation [2]. This organization provides a proper functional barrier to regulate vectorial secretion and intake of molecules. Tight junctions are typically localized at the top third of the region of the cell pole opposite to that in contact with the basement membrane (BM); they seal the intercellular space and establish apical polarity by providing physical segregation between the basolateral and apical domains of the cell membrane. The basal cellular pole is characterized by transmembrane integrin dimers that connect cells to specific extracellular matrix (ECM) molecules of the BM [3].

The presence of specific types of laminins in the ECM environment used for 3D culture has been shown to be critical for the basal polarization of epithelial tissue structures [1,4]. While basal polarity and growth-arrest are routinely used as features of the differentiation of human epithelial structures in 3D culture, the presence of apical polarity is less often emphasized. Studies have acknowledged the presence [5-7] or absence [8,9] of apical tight junctions in breast and colon human epithelial differentiated tissue structures produced in 3D culture and on filters, respectively. However, the culture conditions to replicate the apical pole of the polarity axis have not been investigated.

The need for human epithelial models that replicate the tissue polarity axis, including models of lumen formation with their associated tight junctions, is well illustrated by pathologies in which the alteration of apical polarity is a critical step. Apical polarity loss, as defined by the formation of multilayers of cells or the lack of basal positioning of nuclei, has been used as a parameter for the characterization of early lesions in certain cancerous diseases [10]. When apical polarity organization is altered, as shown by the redistribution of tight junction markers away from apicolateral sites, mammary epithelial cells can be pushed into the cell cycle [7]. This suggests that proper apical polarity is critical for maintenance of epithelial breast tissue homeostasis. Viral and bacterial infections depend on apical polarity for their onset and/or spreading [11,12].

We have used the model of breast acinar differentiation and several types of 3D culture systems to evaluate how the establishment of basoapical polarity is influenced by the extracellular environment. We show that basal polarity and collagen IV contribute to the establishment of apical polarity. Moreover, we are proposing a simplified high-throughput (HTP) culture method to produce basoapically polarized acini from human cells. This method permits direct imaging with low background staining and rapid handling of tissue structures for proteomic and genomic investigations thus, opening new avenues for high-throughput screening on tissue structures.

## Results

### Apical polarity is more sensitive to culture conditions than basal polarity

**Figure 1:**
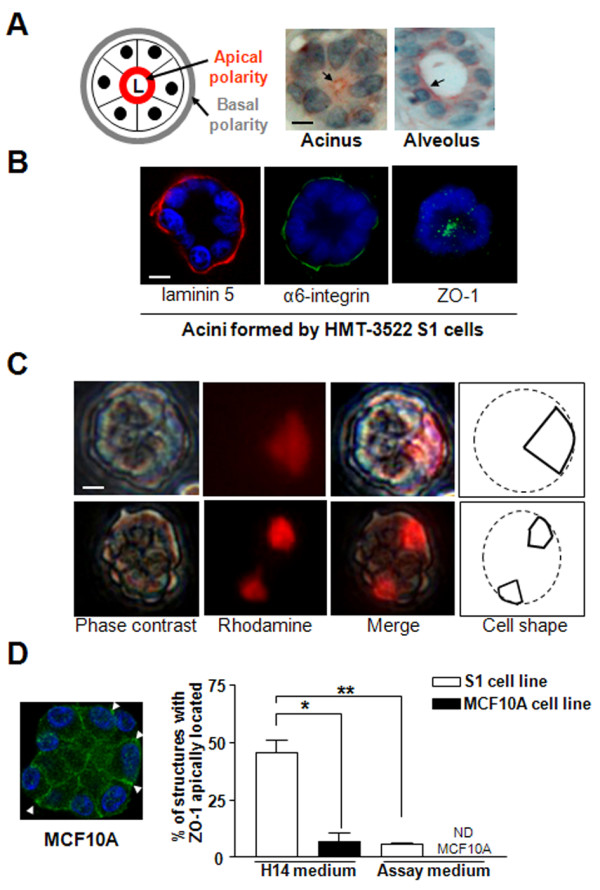
**The formation of differentiated glandular structures depends on the culture medium and cell line**. **A**. Lumen delineated by ZO-1 as shown by immunohistochemistry (reddish-brown) in a mammary acinus and an alveolus from tissue biopsy sections. Hematoxylin staining indicates the position of the nuclei (blue). The drawing indicates the location of basal and apical poles in the glandular structure (nuclei are represented by filled black circles). **B**. Immunostaining for basal (laminin 332) [red], α6-integrin [α6-integrin; green]) and lateroapical (ZO-1 [green]) markers in acini formed by S1 cells in 3D culture. [Note: *in vivo*, the contact between luminal cells and the BM in the mammary gland is discontinuous and occurs where myoepithelial cells are not covering luminal epithelial cells [34]. In contrast, *in vitro*, contact with the BM occurs all along the basal surface of the luminal cells [35], therefore the α6-integrin staining is all around the acinus]. **C**. Microinjection of nondiffusible Rhodamine-Dextran (red) fluorescent dye in individual cells of acini formed by S1 cells in 3D culture. **D**. S1 and MCF10A cells were cultured for 15 days in 3D-Matrigel™ drip either in the H14 medium or the assay medium. Shown is the percentage of multicellular structures with apically polarized ZO-1 (Additional Table S1 with *P *values - see Additional file 1). Also shown is an example of ZO-1 staining in structures lacking apical polarity formed by MCF10A cells in 3D culture (arrowheads point to the basal location of ZO-1 at certain cell-cell junctions). ND = nondetected; size bar, 5 μm; *P < 0.05; **P < 0.01.

The mammary gland is composed of tubuloacinar structures that branch and terminate into acini capable of secreting milk. Resting acini are characterized by a small central lumen (usually less than the size of a cell), whereas secreting acini (or alveoli) have a larger lumen (Figure 1A). A mammary acinus/alveolus is made of an internal and continuous layer of luminal epithelial cells that exert secretory functions and an external and discontinuous layer of myoepithelial cells. Both myoepithelial and luminal epithelial cells make contact with BM components via integrin dimers, while only luminal cells show the presence of apical tight junctions.

The use of one cell type, the epithelial cells, to reproduce acinar differentiation is a simplified yet efficient model to study the function of luminal epithelial cells *in vitro*. The human mammary epithelial cell line, HMT-3522 S1 [13], has been extensively used to study mammary acinar differentiation in 3D culture [1]. They form basoapically polarized acini with tiny lumens when cultured in defined H14 medium [14] (Table 1) in the presence of BM components laminin 111 (formerly laminin 1) and collagen IV-rich gel [15], the Matrigel™ used either in solution in the medium with the cells resting on a gel coat (3D-Matrigel™ drip culture or 'on-top' assay [16]) or as a thick gel in which cells are totally embedded (embedded culture) [1,16] (Additional Methods; Additional Movie 1 - see Additional files 1 and 2). The S1 cell model recapitulates the formation of hemidesmosomes with α6β4 integrins [17] that are critical for the establishment of basal polarity and are observed *in vivo *[3]. Immunostaining for α6- or β4-integrin is routinely used to assess basal polarity in 3D cultures. In addition, a continuous layer of major BM components, like differentiation inducer laminin 332 (formerly laminin 5) (Figure 1B) or collagen IV, should be present at the periphery of the acinus [4,18]. Apical polarity can be assessed by immunostaining for the core tight junction protein Zonula Occludens, ZO-1 [6,8] which, like *in vivo*, should be compartmentalized to the apical side of epithelial structures, against the lumen (Figure 1A and 1B; Additional Movie 2 - see Additional files 1 and 3). An important characteristic of the morphogenesis of glandular structures is the narrow width of the apical pole compared to the basal pole of cells [2]. Microinjection of acinar cells with rhodamine in 3D-Matrigel™ drip cultures revealed a triangular cell shape with the narrowest pole terminating toward the center of the acinus (Figure 1C).

**Table 1:** Comparison between H14 and assay media.

*Composition*	*H14 medium*	Assay medium
**Type of medium**	DMEM/F12	DMEM/F12
**Prolactin**	5 μg/ml	-
**Insulin**	250 ng/ml	10 μg/ml
**Hydrocortisone**	1.4 μg/ml	0.5 μg/ml
**17-Beta-estradiol**	10^-10 ^M	-
**Sodium Selenite**	2.6 ng/ml	-
**Transferrin**	10 μg/ml	-
**EGF**	10 ng/ml	5 ng/ml
**Horse serum**	-	2%
**Cholera toxin**	-	100 ng/ml
**Antibiotics**	-	50 U/ml penicillin 50 μg/ml streptomycin
**% of Matrigel™ drip for 3D culture**	5%	2%

**2D culture process for passages**	culture for 8 to 12 days	culture for 3 to 4 days Medium different from 3D culture medium (5% horse serum, 20 ng/ml EGF)

**Figure 2:**
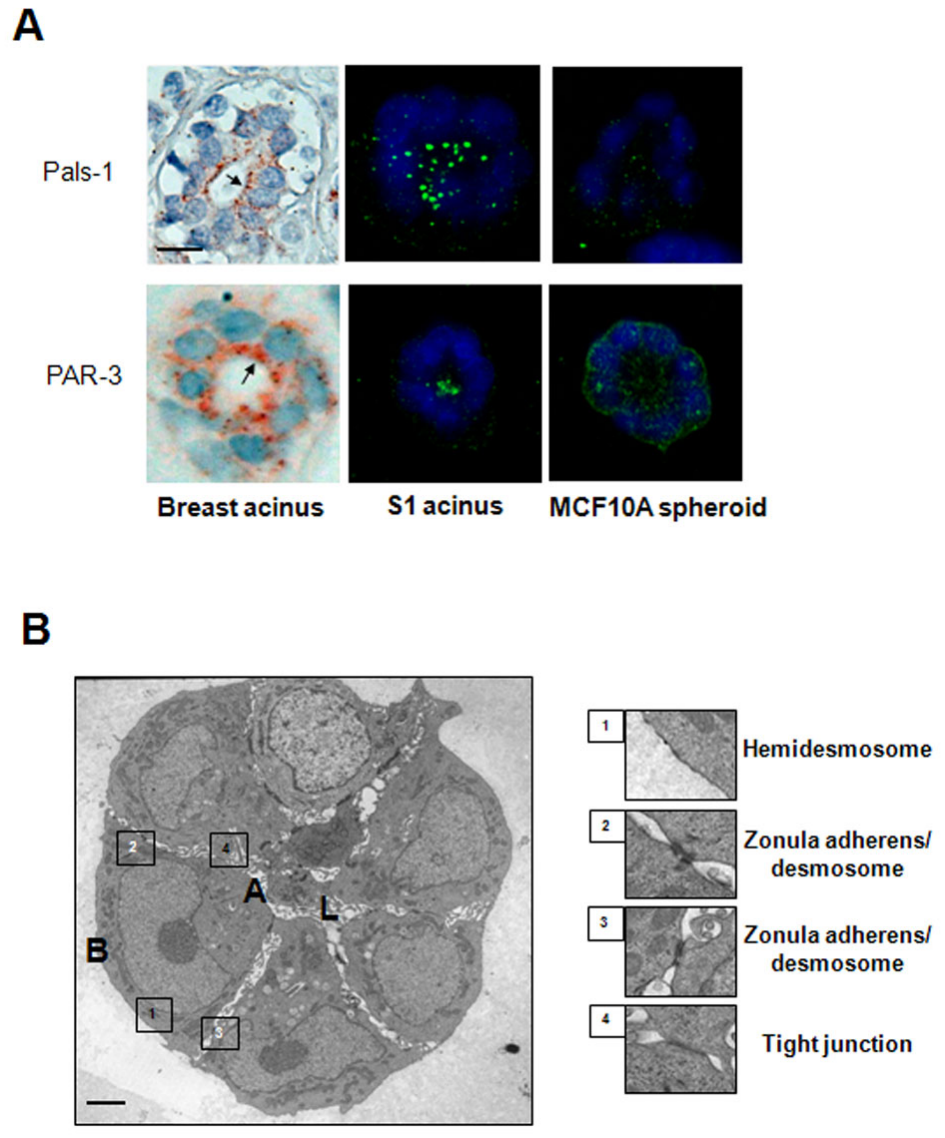
**Apically localized tight junctions in mammary acini**. **A**. Apical concentration of tight junction proteins PALS1 and PAR3 as shown by immunohistochemistry [reddish-brown; arrows] in acini from normal looking mammary tissue (from biopsy sections - left panel), and acini formed by S1 cells in 3D-Matrigel™ drip culture (center panel) as shown by immunofluorescence [green]. Diffuse distribution of PALS1 and PAR3 in multicellular structures formed by MCF10A cells in 3D culture (right panel). Nuclei are counterstained with 4', 6-diamidino-2-phenylindole (DAPI) (blue) in cell cultures and hematoxylin (blue) in IHC. Size bar, 5 μm. **B**. Electron micrograph of a section of an acinus formed by S1 cells in 3D culture. Insets show specific adhesion complexes. Size bar, 1 μm; A = apical; B = basal; L = lumen.

To investigate the importance of the culture conditions in the formation of the basoapical polarity axis, we compared 3D culture conditions of two widely used non-neoplastic breast epithelial cell lines. The H14 medium with 5% 3D-Matrigel™ drip is normally utilized to culture S1 cells to induce acinar differentiation in 10 days [6,16] and the assay medium with 2% 3D-Matrigel™ drip is used with non-neoplastic MCF10A breast epithelial cells to induce differentiation in 15 days [19] (Table 1). More than 93% of acinar structures showed correctly localized BM-component collagen IV, regardless of the culture conditions and cell lines. In contrast, upon 15 days of culture scoring for ZO-1 distribution revealed an approximately 88% increase in S1 acini with loss of apical polarity in the assay medium-2%Matrigel™ drip compared to S1 acini formed in their usual H14 medium-5%3D-Matrigel™ drip. On average 5.3% of the acinus-like structures formed by MCF10A cells showed correct apical polarity in the H14 medium-5%3D-Matrigel™ drip compared to 0% in the assay medium-2%3D-Matrigel™ drip (Figure 1D; Additional Table S1 - see Additional file 1). For the 10-day culture normally used with the H14 medium-5% 3D-Matrigel™ drip condition, the apical location of ZO-1 was still found in a low percentage of MCF10A acini (1%) compared to S1 acini (64.6%). Immunostaining for additional tight junction markers, PAR3 of the PAR3/PAR6/aPKC apical polarity complex and PALS1 of the Crumbs/PALS1/PATJ apical polarity complex [20] showed their concentration to the apical side of cells in normal breast tissue and in the majority of S1 acini, while they were mostly diffusely distributed in the structures formed by MCF10A cells (55.65% and 48.7% of S1 acini had PALS1 and PAR3 apically localized, respectively, compared to 0% of MCF10A multicellular structures) (Figure 2A). Electron microscopy confirmed the presence of apically localized tight junction structures in S1 acini (Figure 2B). Thus, changing the culture conditions, even in the presence of ECM-inducer of differentiation (Matrigel™), dramatically influences apical polarity.

### The type of substratum used for 3D culture affects apical polarization in differentiating cells

To further analyze the importance of culture conditions for phenotypically normal epithelial differentiation, we sought to determine the effect of substrata used in different 3D cell culture systems currently available on the formation of a complete basoapical polarity axis.

**Figure 3:**
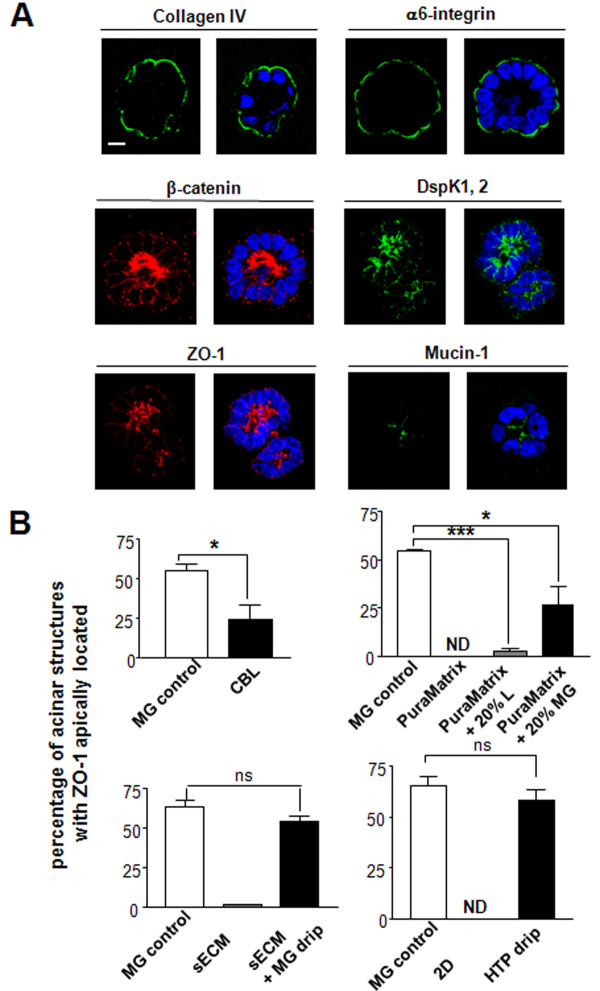
**Distribution of apical polarity marker ZO-1 in acini depends on the substratum for 3D culture**. **A**. Organization of the basoapical polarity axis in acini formed by S1 cells in 3D-Matrigel™ drip cultures as shown by immmunofluorescence staining of basal (collagen IV [green], α6-integrin [green]), lateral [beta-catenin [red] and desmoplakin 1,2 [green]), lateroapical [ZO-1 [red], and apical mucin-1 [green]) polarity markers. Nuclei are counterstained with DAPI (blue). Size bar, 5 μm. **B**. Histograms of the percentages of multicellular structures with apically localized ZO-1 (Additional Table S1 with *P *values - See Additional file 1) when S1 cells are cultured in the presence of chicken basal lamina (CBL) [36], PuraMatrix™, PuraMatrix™ and 20% laminin (PuraMatrix™ +20% L), PuraMatrix™ and 20% Matrigel™ (PuraMatrix™ + 20% MG), synthetic ECM (sECM-drip), synthetic ECM and 5% Matrigel™ drip (sECM + MG drip), monolayer on glass (Two-dimensional-2D), on glass + 5% Matrigel™ drip (High-throughput [HTP] drip) compared to their respective Matrigel™ controls. *P < 0.05; ***P < 0.001; ns = nonsignificant; ND = nondetected (no multicellular structures with apically located ZO-1).

Matrigel™ has been the gold standard for 3D culture of different types of epithelial cells due to its high content (>90%) in differentiation and basal polarity inducing molecules, like laminin 111 [1]. As long as passages are performed according to strict cell culture guidelines (Additional Methods - see Additional file 1), appropriate Matrigel™ lots should reproducibly trigger basoapical polarity in a differentiation capable cell line, as shown by a defined range of basoapical markers (Figure 3A). We first compared extracts of BM obtained from chicken ovary, known to induce the differentiation of ovarian cells [21], to Matrigel™. Chicken basal lamina (CBL) extracts contain laminin 111 and other constituents of the BM (e.g., collagen IV, entactin, and heparan sulfate proteoglycans) [22]. However, in contrast to Matrigel™, CBL originates from normal tissue, and it is not used as a gel; it is used in solution. S1 cells cultured in the presence of CBL extracts formed growth-arrested and basally polarized acini (Additional Figure S1; Additional Table S2 - see Additional file 1) that developed on small focal accumulations of CBL material. Interestingly, no staining could be detected for endogenous collagen IV around acini, and the number of acini with apically localized ZO-1 was significantly lower compared to 3D-Matrigel™ drip control cultures (Figure 3B; Additional Table S1 - see Additional file 1).

An ECM-deprived PuraMatrix™ gel has been successfully used to culture rat hepatocytes in 3D and restore aspects of functional differentiation [23]. This substratum is made of peptides that self-assemble into a 3D hydrogel with a nanometer scale fibrous structure. In contrast to Matrigel™ and CBL obtained from animals, the composition of PuraMatrix™ should not vary. Thirty percent of the population of S1 cells cultured solely within PuraMatrix™ for 10 days was still in the cell cycle, as shown by expression of the cell cycle marker Ki67. In addition, structures formed by S1 cells were devoid of basoapical polarity (Additional Tables S1 and S2; Additional Figure S2 - see Additional file 1). ECM-receptor α6-integrin was found at all cell membranes, collagen IV was mostly nondetectable, and ZO-1 was observed at all cell membranes and within the cytoplasm. Addition of 20% Matrigel™ to PuraMatrix™ restored cell cycle exit but triggered proper collagen IV and α6-integrin distributions in only 32% and 28% of the acini population, respectively (Additional Table S2 - see Additional file 1). Insufficient restoration of basal polarity in the acini population was confirmed with basal polarity marker laminin 332 (54.2% of basally polarized acini in PuraMatrix™ + Matrigel™ cultures compared to 70.2% in Matrigel™-embedded control cultures). Addition of Matrigel™ or laminin 111 to PuraMatrix™ failed to trigger apical polarity, as measured by ZO-1 distribution, in the majority (>68%) of the acini population (Additional Table S1 - see Additional file 1; Figure 3B). Thus, neither the proper localization of basal polarity marker α6-integrin nor the addition of ECM-based differentiation inducers (laminins) is sufficient to produce apical polarity in CBL and PuraMatrix™-based cultures to the same extent as that seen in usual 3D-Matrigel™ drip cultures.

### Development of high-throughput culture for basoapically polarized acini

**Figure 4:**
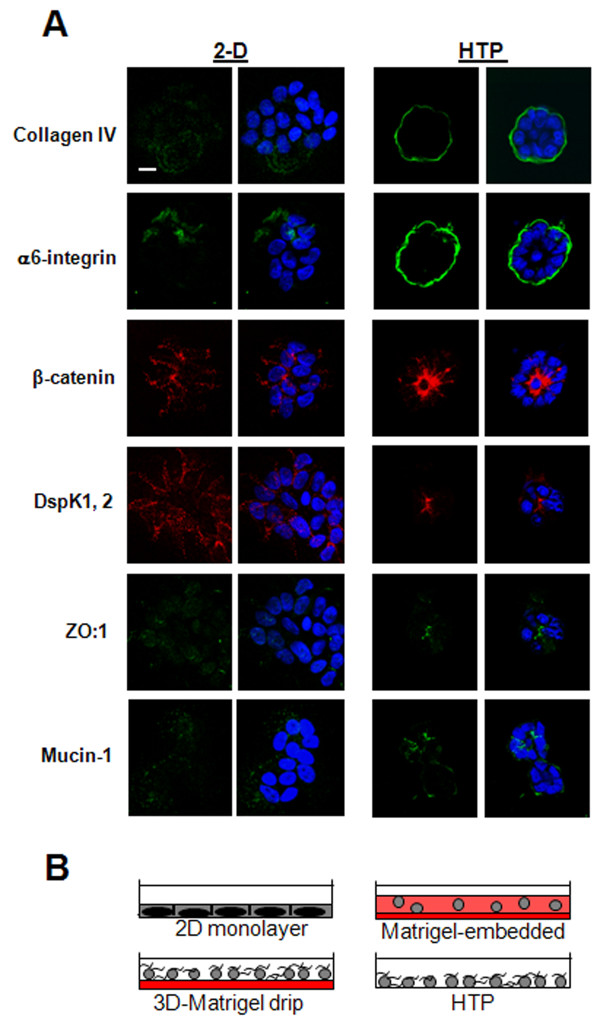
**HTP culture produces a basoapical polarity axis similar to that shown with classical 3D-Matrigel™ cultures**. **A**. Organization of the basoapical polarity axis in 2D and HTP cultures of S1 cells as shown by immmunofluorescence staining of basal (collagen IV [green], α6-integrin [green]), lateral [beta-catenin [red] and desmoplakin 1,2 [red]), lateroapical [ZO-1 [green]), and apical (mucin-1 [green]) polarity markers. Nuclei are counterstained with DAPI (blue). Size bar, 5 μm. **B**. drawings of the different cell culture systems used in this study (2D monolayer; Matrigel™-embedded: cells totally embedded in the Matrigel™ gel; 3D-Matrigel™ drip: 5% Matrigel™ drip on Matrigel™ gel-coated surface; HTP: 5% Matrigel™ drip on glass surface). The gel is represented in red; soluble Matrigel™ dripped in the culture medium is represented by curvy lines.

The substrata used in the previous section are difficult to prepare and/or manipulate and did not promote the formation of the complete polarity axis. Ready-to-use novel substrata based on nanostructural reproduction of an ECM (synthetic ECM or sECM) have been used to trigger spheroid formation with breast tumor cells [24]. We asked whether this sECM might provide a structural framework sufficient to reproduce basoapical polarity. Once plated on sECM, S1 cells formed a flat monolayer with occasional piling up of cells (Additional Figure S3 - see Additional file 1). However, when 5% Matrigel™ was dripped into the culture medium upon plating cells on sECM, acini formed, displaying basal polarity and apical polarity to the same extent as that induced by 3D-Matrigel™ drip culture (Figure 3B; Additional Tables S1 and S2; Additional Figure S3 see Additional file 1). To assess whether sECM played a critical role in induction of polarity upon addition of 5% Matrigel™, we cultured S1 cells directly on glass in the presence or absence of a drip of Matrigel™ in the medium. S1 cells formed a monolayer of cells devoid of polarity in the absence of Matrigel™, whereas adding a drip of 5% Matrigel™ led to the formation of basoapically polarized acini in a percentage similar to that observed in sECM with 5% Matrigel™ drip or usual 3D-Matrigel™ drip cultures (Figures 3B and 4A; Additional Tables S1 and S2; Additional Movie 3 - see Additional files 1 and 4). Thus, the drip of Matrigel™ in the culture medium is sufficient to induce basoapical polarity without precoating the culture surface with a gel.

**Figure 5:**
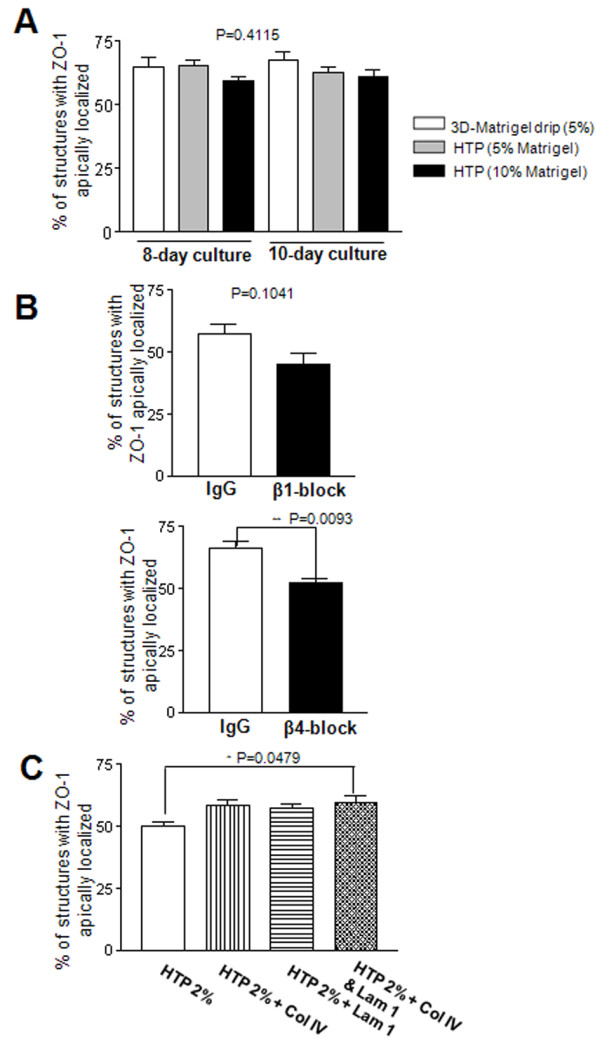
**Both basal polarity and collagen IV contribute to apical polarity in HTP cultures**. **A**. Histogram of the percentages of acini formed by S1 cells (S1 acini) with lateroapical ZO-1 under different culture conditions (5% HTP, 10% HTP, control 5% 3D-Matrigel™ drip for eight and 10 days). **B**. Histograms of the percentages of S1 acini with lateroapical ZO-1, after treatment from day 5 to 8 with function blocking antibody for β1-integrin [β1-block] and β4-integrin [β4-block], and IgG in 5% HTP culture. **C**. Histograms of the percentages of S1 acini with lateroapical ZO-1 after culture in 2% HTP, 2% HTP + collagen IV (Col IV); 2% HTP + laminin 111 (lam 1); 2% HTP + collagen IV (Col IV) and laminin 111 (lam 1), during eight days of culture. *P < 0.05; **P < 0.01.

Three-dimensional-Matrigel™ drip culture normally requires extensive manipulation for set-up and analysis. Ultimately acini have to be removed from the surface of the gel coat for optimal protein and RNA extractions. Moreover, the gel coat creates high background that impairs immunostaining analysis. Therefore, the technique of 5% Matrigel™ without gel coating was further explored for potential use as high-throughput (HTP) culture method. Culture time was shortened by removal of EGF at day 5 of culture instead of day 7, followed by analysis of differentiation efficacy at days 6, 7 and 8; the Matrigel™ concentration was also varied in order to optimize the extent of polarization in the acini population. A Matrigel™ concentration of 20% increased the number of areas with flat monolayers of cells compared to cultures performed with 5 and 10% of Matrigel™ drip. Scoring for basal polarity marker α6-integrin and apical polarity marker ZO-1 showed that 5 and 10% of Matrigel™ drip gave results similar to that obtained with regular 3D-Matrigel™ drip cultures, suggesting that the 5% Matrigel™ drip typically added for 3D-Matrigel™ drip cultures can be used in HTP cultures; (Figure 4B and Figure 5A). Analysis of polarity for each of the culture conditions (5% HTP, 10% HTP, and 3D-Matrigel™ drip) revealed that the duration of cell culture period could be reduced to eight days instead of 10 days (Figure 5A; Additional Figure S4A - see Additional file 1).

Interestingly, the low amount of apically polarized structures in CBL and PuraMatrix™+ Matrigel™ cultures was accompanied with the lack of collagen IV around most multicellular structures. Using the HTP culture we assessed the relative contribution of collagen IV and laminin 111 (inducer of basal polarity) signaling to apical polarity formation (see Additional Methods - see Additional file 1). Blocking signaling via β4-integrin (which binds to laminins 111 and 332 [25]) using function blocking antibodies significantly decreased the number of acini displaying apical polarity compared to nonspecific IgG-treated acini in 5% HTP cultures, suggesting that basal polarity-related signaling triggered upon attachment to the BM was important. Although blocking signaling via β1-integrin (which binds to collagen IV and laminins 111 and 332 [25]) reduced the number of apically polarized acini, this reduction was not significant (Figure 5B). It is important to notice here that our results were obtained starting from acini. In previous work, it was shown that blocking β1-integrin signaling from day 1 of mammary epithelial cell plating, thus prior to acinus formation, led to apoptosis [26]. The difference in these results can be explained by the fact that the predominance and/or function of integrin signaling changes as acinar morphogenesis occurs, notably β4-integrin signaling becomes critical to maintain epithelial homeostasis in acini [18].

To further assess the effect of major molecules responsible for BM integrity on apical polarity formation, we added collagen IV and/or laminin 111 to HTP cultures made from a percentage of Matrigel™ small enough to produce a lower quantity of apically polarized acini compared to control 5% HTP culture. Starting from a 2% HTP culture, a significant increase in the number of apically polarized acini was observed upon addition of a combination of laminin 111 and collagen IV, but the increase was not significant with either molecule added alone (Figure 5C). None of the conditions applied above altered α6-integrin distribution (Additional Figure S4 - see Additional file 1). Adding solely laminin 111 and collagen IV, without the HTP culture conditions, was not sufficient to allow cells to round up and form acini, suggesting that other compounds or conditions present in the Matrigel™ are also necessary for acinar morphogenesis. Based on these results, both basal polarity-directed signaling and collagen IV appear to contribute to apical polarity formation.

## Discussion

The analysis of the different methods of culture of human mammary epithelial cells has enabled us to demonstrate that the development of apical polarity in tissue structures is more sensitive to culture conditions than the production of basal polarity. Such sensitivity might account for the difficulty in visualizing tight junctions at the apical pole of acini. Strains of non-neoplastic cells should be used based on their differentiation capabilities. For instance strains of MCF10A cells that notoriously lack the capability to form apically located tight junctions under usual culture conditions, as shown by fluorescence immunostaining and structural analysis by electron microscopy [8,9], have been used to confirm the critical role of genes, like Crumbs3 in the establishment of apical polarity. Non-neoplastic HMT-3522 S1 cells capable of acquiring basoapical polarity represent a very useful model to develop studies that require well-differentiated tissue structures. With these cells we could identify certain 3D culture conditions necessary for the production of basoapically polarized structures. Notably, by testing different ECM environments we observed that CBL-based 3D culture was not accompanied with the deposition of collagen IV, a BM component critical to stabilize BM integrity [27]. This culture condition might be of interest to better understand the role of collagen IV in epithelial differentiation, notably, as suggested by our data with the HTP culture, in the establishment of apical polarity. The molecular mechanism underlying the lack of apical polarity formation in CBL culture remains to be understood. Possibly, chemical factors necessary for apical polarity formation might be absent from CBL extracts. More excitingly, there is also a possibility that the mechanical environment of the cells might influence apical polarity. Indeed extracellular matrix stiffness was shown to affect the morphogenesis of mammary epithelial cells [28]. It would be interesting to develop an experimental design to specifically assess the effect matrix stiffness on apical polarity.

Our experiments with function blocking antibodies directed toward integrins underscore the importance of the tissue context for the response to ECM alterations. Indeed blocking either β1-integrin or β4-integrin signaling led to differentiation outcomes different from those obtained in previous work [17,26]. This is because such previous experiments were performed before acinar morphogenesis, while our experiments were conducted on already formed acini. These experiments illustrate the impact of tissue architecture resulting from acinar morphogenesis that not only affects the organization of the signaling network [29], but also structural organization all the way to the cell nucleus [30] and thus, influences how cells respond to changes in their microenvironment.

The fact that basoapically polarized tissue structures can be produced relatively rapidly (eight days) and without the gel coat under 5% HTP culture conditions, opens new avenues for high-throughput screenings. With the HTP culture it should become possible to obtain large numbers of polarized tissue structures necessary to perform measurements that require large amounts of cellular material. Importantly, the HTP culture method will be useful, in combination with microscopy techniques, for rapid screening of the effects of environmental factors and drugs on breast epithelium homeostasis (e.g., modulators of apical polarity [31]). The large number of individual tissue structures produced under these conditions will allow for meaningful statistical analyses. This type of analysis was previously impaired by the thickness of the culture due to the gel coat and/or the nonspecific background signals due to the gel. A simplified method to produce glandular tissue structures should lead to the implementation and/or development of novel tests to help answer biological and medical questions.

## Conclusion

Our findings demonstrate an important role for the BM in the establishment of apical polarity, suggesting that any extracellular factor that perturbs either cell-BM connections or the stability of the BM (via collagen IV) would rapidly affect the functional integrity of the epithelium and possibly its homeostasis since apical polarity proteins have been linked to proliferation control and cancer development [32]. Due to the prominent role of apical polarity in early stages of cancer development, it is paramount to have a model that permits the identification of factors that control such polarity. We believe that the HTP culture developed here for breast epithelial cells could provide an important tool for high content screening of risk and protective factors of epithelial architecture and thus, homeostasis.

## Methods

### Cell culture

Non-neoplastic human mammary HMT-3522 S1 [13] and MCF10A [33] epithelial cells were cultured at 37°C in 5% CO_2_, either in chemically defined DMEMD/F12-based H14 medium [14] or in assay medium [19] (Table 1) changed every two to three days.

For regular 3D assays including 3D-Matrigel™ drip, and cells embedded in Matrigel™ (BD Biosciences, Bedford, MA) and PuraMatrix™ (BD Biosciences), S1 cells were cultured for 10 days in H14 medium (Additional Methods - see Additional file 1). Unless otherwise specified, at day 7 (or day 8), cells were induced to exit the cell cycle upon culture without addition of epidermal growth factor (EGF) during 72 (or 48) hours. Important considerations to maintain full differentiation capabilities of the S1 cell line: Firstly, immortalized non-neoplastic cells are a heterogeneous population in which some cells have better differentiation capabilities than others. In order to keep a high percentage of differentiation-capable cells, a defined concentration of cells is used for plating for monolayer and 3D cultures and cells are kept in culture for 10 passages maximum, with passage 60 as the latest possible passage used for 3D culture assays. Secondly, for propagation of cells as a monolayer (i.e., without addition of exogenous substratum), passages performed in cell culture flasks occur between eight to 12 days of culture, which usually corresponds to 60 to 70% cell confluence. If cells are detached from the surface of the flask too early, the population will be enriched in cells that had attached rapidly, which might ultimately impair proper acini formation.

For HTP cultures, 50,000 S1 cells were plated per well of glass bottom 4-well chamber slides in 200 μl of H14 medium. After five minutes, 200 μl of H14 medium containing 4, 10, 20 or 40% Matrigel™ were added to each well in drops all over the cell culture surface bringing Matrigel™ to a final concentration of 2, 5, 10 or 20%, respectively. It is important that the final volume of culture medium containing cells is not actively mixed or shaken in order to allow Matrigel™ components to deposit slowly and evenly at the bottom of the well and on the cells. The first H14 medium change took place after 48 hours of culture without addition of Matrigel™ in the fresh medium. EGF was omitted from the culture medium 48 to 72 hours prior to the end of the culture period. Similar conditions were used for 3D culture with sECM (Ultra-Web™ synthetic surface, Corning Inc., Lowell, MA, USA) with the following specifications. The sECM coverslips were exposed to UV light for 30 minutes to sterilize them before use. After placing the coverslips at the bottom of a 12 well-plate, cells were plated at a density of 89,000 cells/well, and cultured in H14 medium supplemented with 1× penicillin/streptomycin (from 100× stock containing 5000 units of penicillin and 5000 μg/ml of streptomycin, Invitrogen, Carlsbad, CA, USA).

### Immunofluorescence analysis

Immunostaining was performed as described previously [6], on cryosections (Additional Methods - see Additional file 1) for cells embedded in Matrigel™ and PuraMatrix™-based substrata, or directly on sECM coverslips and in 4-well chamber slides uncoated, or coated with Matrigel™ or CBL.

### Statistical analysis

Data are presented as means ± SEM. Statistical comparisons were performed using GraphPad Prism 3.0 software (GraphPad Software Inc, San Diego, CA, USA). Nonpaired *t*-test was used for comparison of two groups, and one-way Anova test for comparison of more than two groups. A *P *< 0.05 was considered significant.

## Abbreviations

2D: two-dimensional; 3D: three-dimensional; BM: basement membrane; CBL: chicken basal lamina; ECM: extracellular matrix; DAPI: 4', 6-diamidino-2-phenylindole, EGF: epidermal growth factor; HTP: high-throughput; sECM: synthetic extracellular matrix, ZO: zonula occludens.

## Authors' contributions

CP carried out experiments with different extracellular substrata for 3D cell culture and drafted the manuscript; LC carried out experiments related to the comparison of apical polarity status in two mammary epithelial cell lines and with human tissue, and to the comparison of HTP culture conditions, and participated in the making of the draft of the manuscript; HA carried out the PuraMatrix™-based culture experiments and immunostaining and scoring of certain culture conditions involving different substrata, was involved in experiments related to microinjection, and participated in the making of the draft of the manuscript; LW carried out experiments related to the study of basal polarity and collagen IV in HTP cultures; AU carried out the microinjection experiments in 3D culture; JS carried out the imaging of immunostained cultures and produced the movies included in the manuscript; EKA provided expertise for the experiments with chicken basal lamina and participated in the critical writing of the manuscript; SAL conceived the study, and participated in its design and coordination and in the analysis of the results, helped draft the manuscript, and finalized the writing of the manuscript. All authors read and approved the final manuscript.

## Supplementary Material

Additional file 1 **Additional information**. This file includes, in order, additional methods, additional table S1 (Table S1), additional table S2 (Table S2), additional figure S1 (Figure S1), additional figure S2 (Figure S2), additional figure S3 (Figure S3), additional figure S4 (Figure S4), movie legends). Click here for file

Additional file 2 **Movie 1**. 3D reconstruction of the lumen in S1 cells in 3D-Matrigel drip culture. Click here for file

Additional file 3 **Movie 2**. 3D reconstruction of an acinus in S1 cells in 3D-Matrigel drip culture Click here for file

Additional file 4 **Movie 3**. 3D reconstruction of an acinus formed by S1 cells in HTP culture. Click here for file
