# Supplementary material for: Factors necessary to produce basoapical polarity in human glandular epithelium formed in conventional and high-throughput three-dimensional culture: example of the breast epithelium
Source: BMC Biol. 2009 Nov 16;7:77. doi: 10.1186/1741-7007-7-77 (PMC2784453; doi:10.1186/1741-7007-7-77)
Supplement: Additional file 1 — Additional information. This file includes, in order, additional methods, additional table S1 (Table S1), additional table S2 (Table S2), additional figure S1 (Figure S1), additional figure S2 (Figure S2), additional figure S3 (Figure S3), additional figure S4 (Figure S4), movie legends). [file 1741-7007-7-77-S1.PDF]

Additional methods

Antibodies used for immunostaining

Mouse monoclonal antibodies against mucin-1 [0.56 µg/ml] (clone DF3, DAKO, Carpinteria, CA), were used on cells fixed in 2% paraformaldehyde. Mouse monoclonal antibodies against β-catenin [2.5 µg/ml] (clone 14, BD Biosciences), ZO-1 [5 µg/ml] (Zymed, San Francisco, CA), Ki67 [4.8 µg/ml] (clone Ki67, DAKO), collagen IV [1.6 µg/ml] (clone CIV 22, DAKO), desmoplakin 1&2 [5 µg/ml] (MP Biomedicals, Aurora, OH), laminin 332 (laminin 5) [1/100, which was ~10 µg/ml] (a kind gift from Dr. Peter Marinkovich, Stanford University, CA), rat polyclonal antibodies against α6-integrin [10 µg/ml] (clone NKI-GoH3, Chemicon), and rabbit polyclonal antibodies against PALS1 [1/100] (Upstate/Millipore, Billerica, MA), PAR3 [13.3 µg/ml] (Upstate), ZO-1 [2.5 µ,g/ml] (Zymed) and KI-67 [1/1500] (Novocastra Laboratories, Newcastle upon Tyne, UK) were used on cells incubated in permeabilization buffer (0.5% triton X-100, 100 mM NaCl, 300 mM sucrose, 10 mM pipes, pH 6.8, 5 mM $MgCl_2$, 1 mM pefabloc, 10 µg/ml aprotinin, 250 µM NaF), prior to fixation in 4% paraformaldehyde. Secondary antibodies were TexasRed® or FITC dye conjugated affinipure F(ab')2 Fragment donkey anti-Rabbit [6 µg/ml] (Jackson ImmunoResearch, West Grove, PA), FITC dye conjugated affinipure F(ab')2 Fragment donkey anti-Rat [6 µg/ml] (Jackson ImmunoResearch), Alexa Fluor® 488, Alexa Fluor® 594, or Alexa Fluor® 568 F(ab')2 fragment Goat anti-Mouse [6.67 µg/ml] (Molecular Probes, Eugene, OR). For all immunostainings DNA was counterstained with 4', 6-diamidino-2-phenylindole (DAPI) [0.5 µg/ml] and samples were mounted in ProLong anti-fade solution (Molecular Probes).

3D culture in the presence of Matrigel™

S1 cells were induced to recapitulate the formation of polarized glandular structures (acini) upon culture in the presence Matrigel™ (BD Biosciences, Bedford, MA) [1, 31]. A 3D-Matrigel "drip" method [6] was used for cultures in 4-well chamber slide (Nalge Nunc International, Naperville, IL). Briefly, S1 cells (50,000 cells/well) were plated on surfaces precoated with 60 µl Matrigel™/well 30 min earlier. Then, H14 medium containing 10% Matrigel™ was carefully dripped over the entire culture surface. Medium was changed every two to three days, without further addition of Matrigel™ until completion of acinar differentiation. Embedded culture in Matrigel™ was performed in 4-well plates (Nalge Nunc International). Briefly, for each well 250,000 S1 cells were resuspended in 30 µl of DMEM/F12 medium before gentle mixing (by pipetting up and down a couple of times) with 300 µl of ice-cold Matrigel™. The mixture was quickly deposited drop by drop in one well of the 4-well plate precoated 30 min earlier with 20 µl of Matrigel™. After 30 min incubation at 37˚C, 500 µl of H14 medium was added to each well. Cell culture proceeded for 10 days with H14 medium change every two to three days.

3D culture in PuraMatrix-peptide hydrogel

PuraMatrix-peptide hydrogel was used according to the supplier's instructions (BD Biosciences). Briefly, for each well of a 4-well plate, 250,000 cells were washed in DMEM/F12 cell culture medium containing 10% sucrose and pelleted by centrifugation at 157g for 5 min. Cells were resuspended in 175 µl DMEM/F12 with 10% sucrose and mixed with 175 µl of 0.1% PuraMatrix. The PuraMatrix-cells mixture was immediately

deposited into a well precoated with 0.01% Poly-L-Lysine solution (Sigma). Poly-L-Lysine plates were prepared according to the manufacturer's instructions. PuraMatrix-cells mixtures were incubated in H14 medium for 5 min at 37˚C, after which the culture medium was replaced by fresh H14 medium. The mixture was incubated at 37˚C for 30 min and the H14 medium was changed twice during this period (these media changes were performed to counteract the low pH of the hydrogel). For experiments using a mixture of PuraMatrix and laminin 1 or PuraMatrix and Matrigel^{TM}, cells were washed as previously described and resuspended in 140 µl of DMEM/F12 with 10% sucrose and 70 µl of laminin (667 µg/ml stock solution) (BD Biosciences) or Matrigel^{TM} (9.21 mg/ml stock solution) (BD Biosciences). The mixture was transferred into an eppendorf tube containing 140 µl 0.1% PuraMatrix. The final concentration of laminin was 20% (v/v) corresponding to 133.4 µg/ml (w/v). The final concentration of Matrigel^{TM} was 20% (v/v) corresponding to 1.84 mg/ml (w/v). The mixtures were immediately transferred into wells of 4-well plates precoated with Poly-L-Lysine and washed a few times with fresh H14 culture medium as described above.

3D culture in the presence basal lamina from chicken granulosa cells

Basal lamina was isolated from the largest preovulatory follicle (F1) of Single Comb White Leghorn hens in their first year of reproductive activity as described earlier [36], under PACUC approval # 89-055-04. Briefly, the granulosa layer was separated from the thecal layer using forceps and placed in hypotonic solution. Granulosa cells, sandwiched between the basal lamina and the perivitelline layer were lysed and the basal lamina and the perivitelline layer were separated. Basal laminae were solubilized

in buffer containing 6 M guanidine-HCL, 50 mM Tris-HCL pH 7.4, and 5 mM β-mercaptoethanol, and agitated for 60 min at 4ºC. Solubilized basal laminae were placed in a 3-kDa cutoff dialysis membrane and dialyzed against 150mM NaCl and 50mM Tris-HCL pH 7.4.

Solubilized chicken basal lamina (CBL) was deposited in 4-well chamber slides (144 µg of basal lamina proteins/well) and dried overnight at room temperature. S1 cells (50,000 cells/well) were plated on CBL precoated surfaces and received a drip of H14 medium containing 5% solubilized CBL (v/v) at the time of plating as described for the 3D-Matrigel drip technique. 1X penicilline-streptomycin (from 100X stock containing 5000 units of penicilin and 5000 µg/ml of streptomycin, Invitrogen), was added to the culture medium.

HTP cultures with laminin 111 and/or collagen IV and function blocking antibodies against integrins

S1 cells were cultured with the HTP method (see material and methods) for eight days in the presence of 2% Matrigel drip, or in the presence of 2% Matrigel^{TM} mixed with 20% laminin 111 (see section on culture in PuraMatrix above) and/or with 20 µg/ml collagen IV (BD Biosciences). For the function blocking experiments, S1 cells were cultured in 5% HTP. At day 5 of culture cells were incubated either with 15 µg/ml of function blocking anti-β1 integrin antibody (AIIB2 clone, a kind gift from Dr. Mina Bissell), or 15 µg/ml of function blocking anti-β4 integrin antibody (Chemicon), or 15 µg/ml IgG (Jackson Immunoresearch) for another three days in 5% HTP.

Preparation of gels for cryosectioning

3D cell cultures embedded in Matrigel™ and PuraMatrix were prepared for cryosectioning by removal of H14 medium followed by 15 min incubations in increasing concentrations of sucrose (18% and 30%, respectively) prior to mounting in Tissue-Tek freezing solution (Sakura, Torrance, CA). Mixture of 3D cultures and Tissue-Tek were frozen by placing the 4-well plate on dry-ice for 30 min. Cell culture blocks were removed by gentle twists using a tweezer, sectioned in a cryostat with a thickness setting of 20 μM, and stored at -80ºC until immunofluorescence labeling.

Microinjection of acini

Individual cells within acini were microinjected with 2% Rhodamine-B isothiocyanate-Dextran (RD, Sigma-Aldrich) dissolved in 0.15M LiCl. Injection was performed ionophoretically with hyperpolarizing pulses of 4-5.5nA/500ms at a frequency of 0.75 Hz for 1 min, using a Grass S88 stimulator (Grass Instruments, West Warwick, RI). The micropipettes utilized for the injection were made from borosilicate capillary tubing (1.0 mm OD X 0.75 mm ID/Fiber; Frederick Haer & Co, Bowdoinham, ME) using a P-97 microelectrode puller (Sutter Instruments; Novato, CA). The sharpness of the micropipettes was estimated by measuring tip resistance upon filling with 3M KCl; resistance was 15-20 MOhm. Penetration through the cell membrane was determined by monitoring the membrane potential, which ranged between -19 and -35 mV, using an AxoClamp 2B amplifier (Axon Instruments, Foster City, CA). During microinjections, cells were kept in culture medium at room temperature. Epifluorescence microscopy

was used to image acini with the microinjected cells marked by nondiffusible RD (red fluorescence).

Immunohistochemistry

Archival formalin-fixed normal adult breast tissues were obtained from the Department of Surgical Pathology at the Indiana School of Medicine, Indianapolis, IN. Tissue samples were used according to Institutional Review Board approval # 0502000712. Tissue sections were sequentially deparaffinized in xylene and rehydrated in increasing percentages of ethanol. Antigen retrieval was done by boiling the slides in Target Retrieval Solution (Dako Cytomation) for 10 min. Following washes in TBS, slides were incubated with 3% Hydrogen Peroxide in methanol for 15 min to block endogenous peroxidase. Tissues were then washed in TBS followed by avidin and biotin blocking (Vector Laboratories, Burlingame, CA) 15 min each. Slides were blocked 1h with blocking reagent (TSA biotin system, PerkinElmer Life Sciences, Boston, MA) prior to overnight incubation with rabbit polyclonal anti-ZO-1 (5 µg/ml), rabbit polyclonal anti-PAR-3 (10 µg/ml, Upstate), Pals-1 (1/100, Upstate). The next day, slides were washed with TBS and TBST (TBS with 0.05% Tween-20) before incubating with secondary biotinylated goat anti-rabbit (0.99 µg/ml, DakoCytomation) or biotinylated horse anti-mouse (1.5 µg/ml, Vector Laboratories) immunoglobulins, for 1h. Following washes with TBS and TBST, samples were incubated for 30 min with streptavidin-horseradish peroxidase diluted 1/100 in blocking reagent (PerkinElmer Life Sciences). Tissues were washed in TBS and TBST prior to incubation with biotinyl-tiramide signal amplification for 15 min (PerkinElmer Life Sciences). Final incubation with streptavidin-horseradish

peroxidase was done after TBS and TBST washes. Aminoethyl Carbazol (AEC) substrate (Zymed) was used to develop the staining and nuclei were counterstained with hematoxylin (Richard-Allan Scientific, Kalamazoo, MI). Finally, slides were covered with Clearmount (Zymed) and let stand overnight before mounting with Permount mounting reagent (Fisher Scientific, Fair Lawn, NJ).

Electron microscopy

Cells were cultured in the presence of Matrigel^{TM} (3D-Matrigel drip method) in a 4-well chamber slide for 10 days to produce acini. Cells were fixed in 3% paraformaldehyde/0.1% glutaraldehyde *in situ*. Then, the chamber of the slide was removed and the acini/Matrigel mixture from each well was gently scraped from the slide into an eppendorf tube and centrifuged. Pellets were post-fixed with 0.5% aqueous OsO_4 and embedded in LX-112 resin mixture. Blocks were thin-sectioned and stained for 5 min in 2% uranyl acetate. Samples were imaged with a FEI/Philips CM-10 Biotwin transmission electron microscope (FEI Company, Hillsboro, OR) using 80kV accelerating voltage. Images were captured on Kodak Electron Image SO-163Film.

Scoring process

Scoring of basoapical polarity markers was performed according to specific guidelines established for each marker. Most of the experiments were scored twice by two different people and/or using coded slides so that the treatment or culture conditions remained unknown to the scorers. Basal polarity markers were considered well organized if they were continuously surrounding the acinar structures (see Supplementary Table 2 for

representative drawings) and the apical polarity marker ZO-1 was considered well organized if it was concentrated into spots or patches towards the center of the acinar structures (see Supplementary Table 1 for representative drawings). A minimum of 100 acinar structures per marker and per biological replicate (i.e., specific culture set) or the entire culture device (when the substratum used did not permit the formation of many three-dimensional structures like in PuraMatrix gels) were scored. A minimum of three biological replicates was used for scoring unless stated otherwise.

Image acquisition and movies

Images were acquired using a Radiance 2100 MP Rainbow (Bio-Rad, Hemel Hempstead, England) on a TE2000 (Nikon, Tokyo, Japan) inverted microscope using a 60x oil 1.4 NA lens. Red (Alexa 568), FITC and DAPI fluorescence images were collected sequentially to avoid any possible bleed through. The Alexa 568 was excited at 543 nm using the green HeNe laser and fluorescence emission greater than 560 nm in wavelength was collected. Then, FITC was excited with the 488 nm line of the 4-line argon and the emission was collected with a 500LP, 550SP filter combination. Multi-photon excitation for the DAPI was provided by the Mai Tai laser (Spectra-Physics, Mountain View, CA) at 750 nm and the emission between 420 and 480 nm was collected. For each area of interest, a single plane through the center or a z-series through the entire depth of the acinus was acquired with LaserSharp 2000 software (Bio-Rad). This software was used to create movies displaying each image of the z-series with all three colors. Additionally, movies of acini rotating were created using maximum value projections. All movies were then processed to display the

individual color channels and compressed using MediaStudio (Ulead Systems, Torrance, CA). Volume rendering of the 3-color channel data was performed using VoxelView software (Vital Images, Inc., <color r=0100 g=0100 b=0100>Minnetonka, MN). This software was also used to create a model of the cavity within the acinus by tracing the border of the cavity.

	Acini with strictly apical ZO-1	P value	N=
**S1 in H14 medium 15days**	41.23% ± 6.11		3
**MCF10A in H14 medium 15 days**	5.27% ± 5.22	0.066<br>**	3
**S1 in assay medium 15days**	4.83% ± 4.83	0.0033<br>**	3
**MCF10A in assay medium 15days**	0%		3
**S1 in H14 medium 10 days**	64.6% ± 1.45		2
**MCF10a in H14 medium 10 days**	1% ± 1		2
**CBL culture**	23.93% ± 9.36	0.0387<br>*	3
**Matrigel control**	55.1% ± 4.25		3
**PuraMatrix**	0%		3
**PuraMatrix + 20% Laminin I**	2.62% ±1.03	<0.001<br>***	3
**PuraMatrix + 20% Matrigel**	31.85% ± 9.74	0.0275<br>*	3
**Matrigel control**	54.37% ± 0.7		3
**sECM**	1.57% ± 0.57		3
**sECM + 5% Matrigel**	54.43% ± 3.22	0.1791	3
**Matrigel control**	63.23% ± 4.35		3
**2D**	0%		3
**HTP + 5% Matrigel**	58% ± 5.19	0.3542	3
**Matrigel control**	65.23% ± 4.56		3
Representative ZO-1 staining patterns (in grey)in an acinus Empty circles = nucleus L = lumen	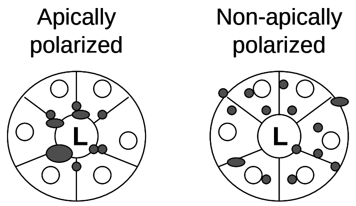		

Table S1. Percentage of multicellular structures with apically localized ZO-1 under different culture conditions. First column: Culture conditions. Second column: percentage ± SEM of acini with ZO-1 apically localized. Third column: p value of nonpaired t-test comparison (each culture condition was compared to S1 in H14 medium [first six rows]; each treatment was compared to its respective Matrigel control [last 12 rows]). Fourth column: number of independent experimental replicates. The drawing at the bottom of the table indicates criteria used to score the lateroapical localization of ZO-1. *p<0.05; **p<0.01; ***p<0.001

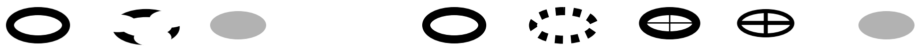

	Collagen IV				α6-integrin					Ki67
**Matrigel (control)**	**80**	14	4	2	**85**	**5**	9	1	0	≤5
**CBL (3D)**	**1**	0	0	99	**61**	**31**	0	0	9	≤5
**PuraMatrix**	**0**	0	3	97	**0**	**5**	8	87	1	30
**PuraMatrix + Matrigel**	**32**	50	15	4	**21**	**7**	54	18	0	≤5
**sECM (2D)**	**0**	0	0	100	**0**	**2**	0	0	97	≤5
**sECM + Matrigel**	**79**	18	2	1	**77**	**9**	0	0	15	≤5
**2D monolayer**	**0**	2	0	98	**0**	**7**	0	0	94	≤5
**HTP-drip**	**86**	14	1	0	**86**	**10**	0	0	4	≤5
		Altered distribution					Altered distribution			

Table S2. Scoring of basal polarity markers under different culture conditions. S1 cells were cultured for 10 days under the different conditions listed in the first column of the table. Immunostaining for collagen IV and α6-integrin was scored according to the distribution patterns drawn above the respective columns. Averaged percentages corresponding to patterns considered as normal are in bold and the conditions that gave above 60% acini correctly basally polarized are shaded in gray. The last column in the table indicates the average percentage of cells in the cell cycle as measured by Ki67 positive staining in multicellular 3D structures (except with sECM and 2D cultures for which scoring was done on flat monolayers of cells). Scoring was done for at least three independent cultures.

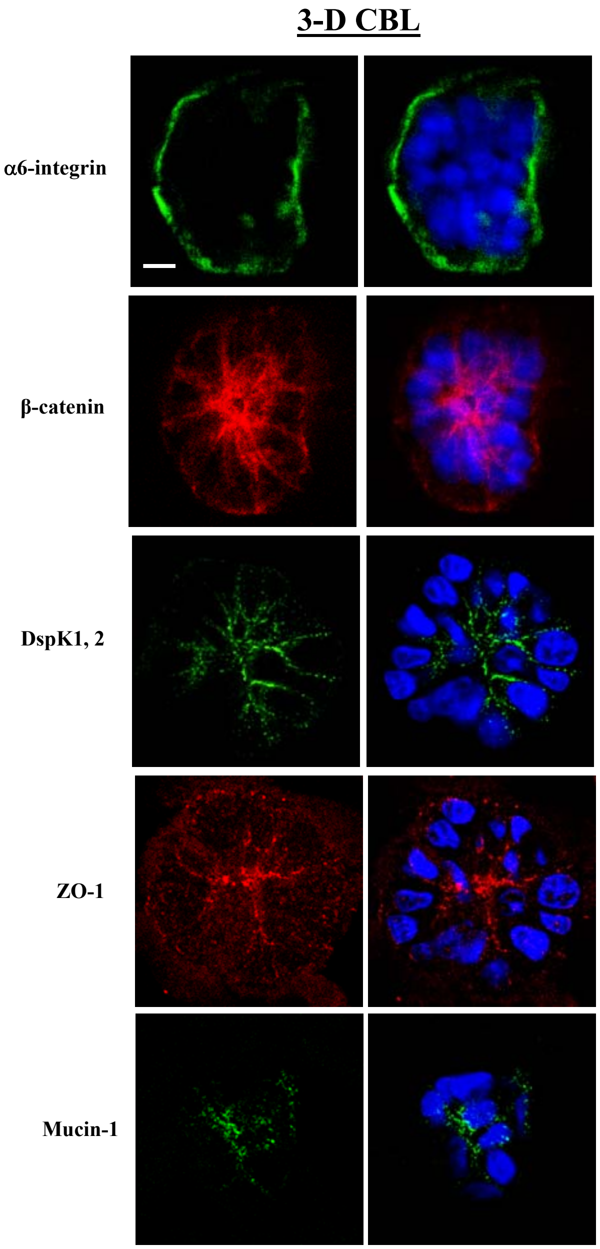

<u>Figure S1</u>: Differentiation parameters in CBL-based cultures of S1 cells. Organization of the basoapical polarity axis as shown by immmunofluorescence staining of basal (α6-integrin [green]), lateral [β-catenin [red] and desmoplakin 1,2 [green]), lateroapical [ZO-1 [red], and apical mucin-1 [green]) polarity markers. Nuclei are counterstained with DAPI (blue). Size bar, 5 μm

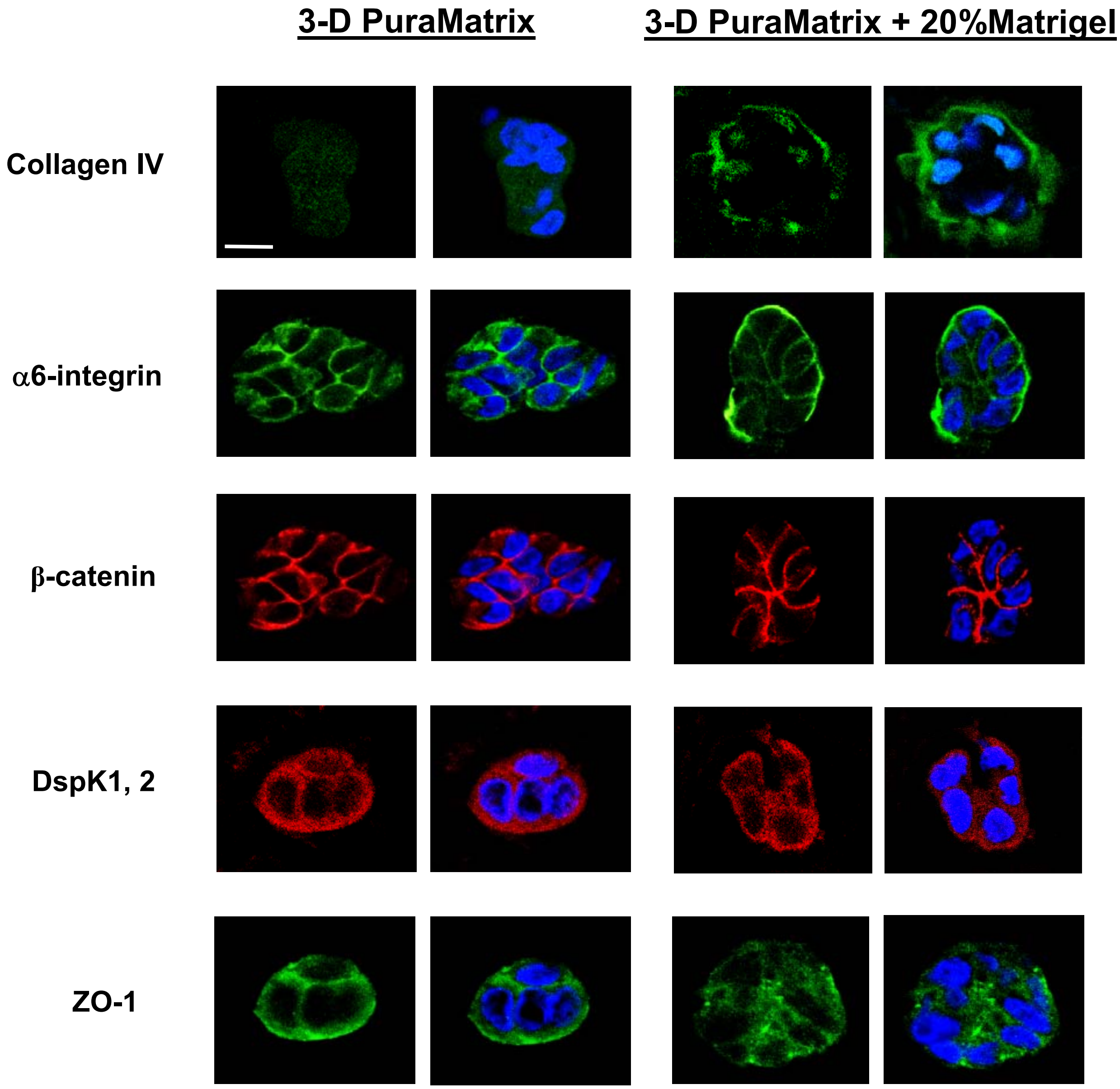

Figure S2: Differentiation parameters in PuraMatrix-based cultures of S1 cells. Organization of the basoapical polarity axis as shown by immmunofluorescence staining of basal (collagen IV [green] and α6-integrin [green]), lateral [β-catenin [red] and desmoplakin 1,2 [red), lateroapical [ZO-1 [green] polarity markers in multicellular structures formed in PuraMatrix and PuraMatrix + 20% Matrigel cultures. Nuclei are counterstained with DAPI (blue). Size bar, 5 µm.

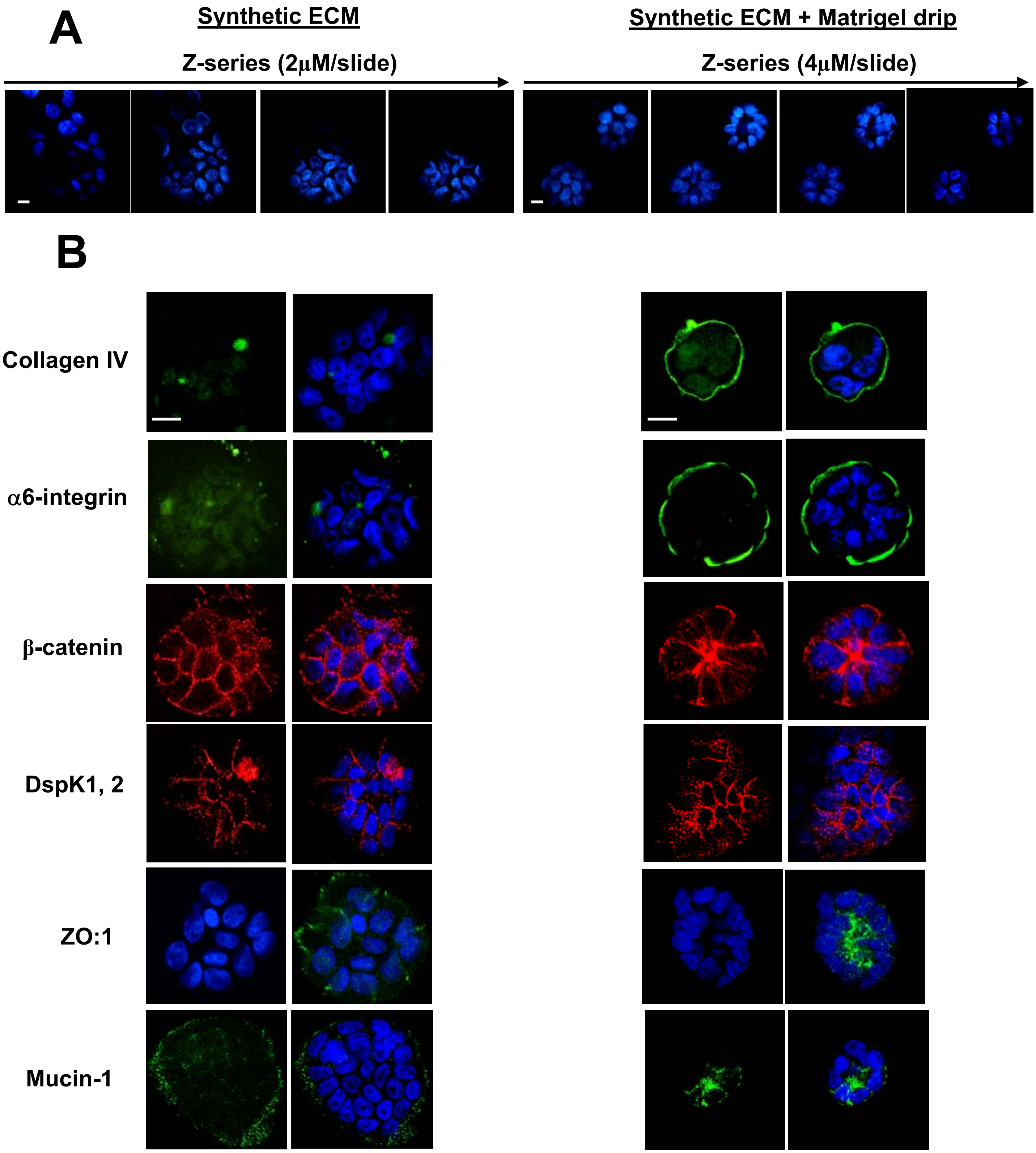

Figure S3: Differentiation parameters in synthetic ECM (sECM)-based cultures of S1 cells. **A.** Z-series showing a multilayered area of cells on sECM alone (left panel) and on sECM + 5% Matrigel drip (right panel). **B.** Organization of the basoapical polarity axis as shown by immmunofluorescence staining of basal collagen IV [green] and α6-integrin [green], lateral [β-catenin [red] and desmoplakin 1,2 [red]), lateroapical [ZO-1 [green], and apical mucin-1 [green]) polarity markers in multicellular structures in sECM cultures (left panel) and sECM + Matrigel drip cultures (right panel). Nuclei are counterstained with DAPI (blue). Size bar, 5 μm.

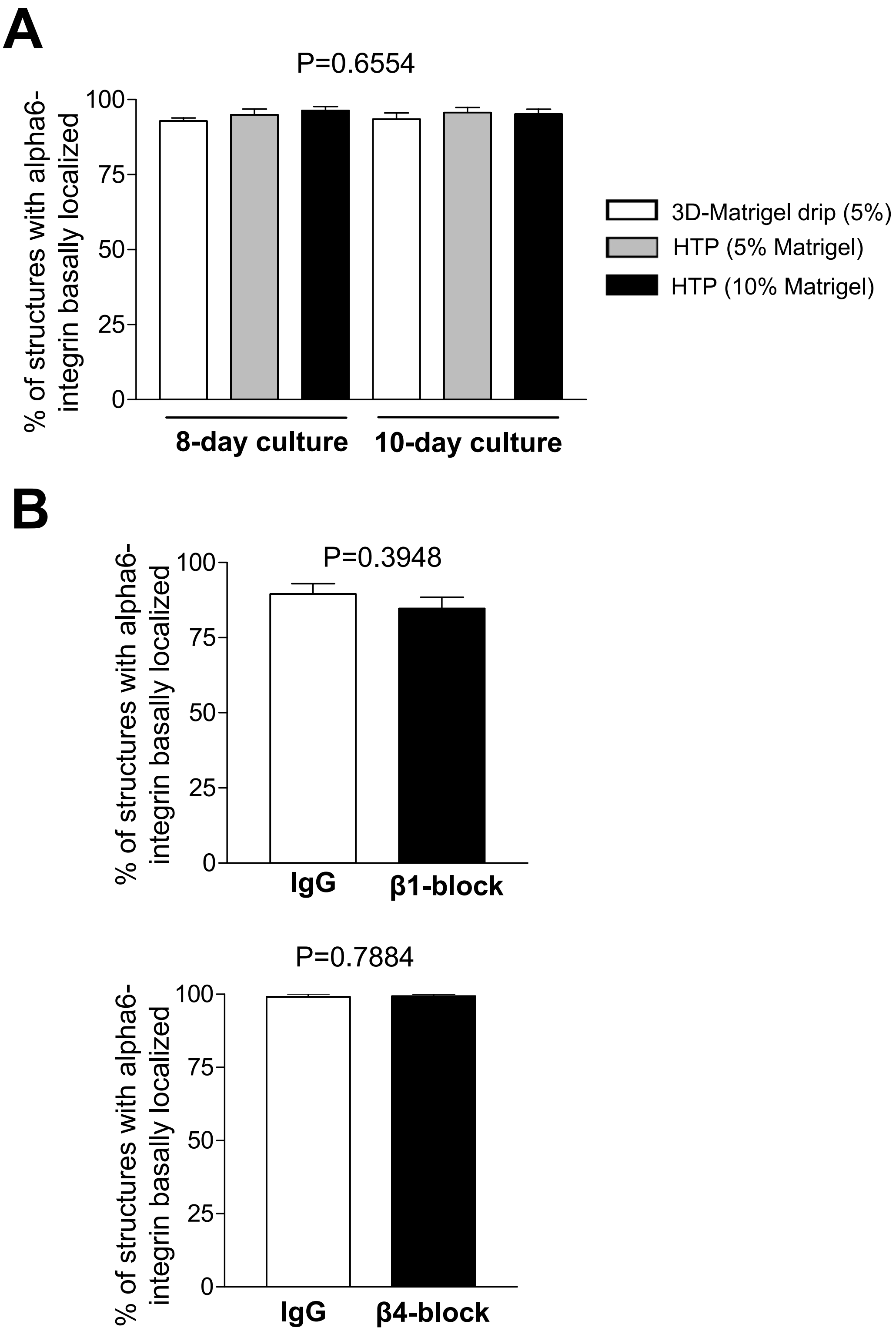

<u>Figure S4</u>: The basal distribution of α6-integrin is not significantly altered by the percentage of Matrigel in the HTP culture of S1 cells or by function-blocking antibodies against β1- and β4-integrins A. Histogram of the percentages of S1 acini with basal α6-integrin under different culture conditions (5% HTP, 10% HTP, control 5% 3D-Matrigel drip for 8 and 10 days). **B.** Histograms of the percentages of S1 acini with basal α6-integrin, after treatment from day 5 to 8 with function blocking antibody for β1-integrin [β1-block] and β4-integrin [β4-block], and IgG in 5% HTP culture; n = 3.

Movie legends

Movie 1: **3D reconstruction of the lumen in S1 cells in 3D-**Matrigel drip culture

A model of the cavity within the acinus formed by S1 cells was created by tracing the border of the cavity (see Supplementary Methods). This model is displayed in yellow in the reconstructions. In the volume rendered movie, the portion of the volume nearest the viewer is cut away to reveal the cavity as the volume rotates about its axis. The reconstruction of the lumen (in yellow) is based on triple staining for the cell nucleus (DNA in blue), cell-cell contact (beta-catenin in red) and apical region (ZO-1 in green). ZO-1 staining is not seen due to the yellow model of the lumen overlapping with ZO-1 staining.

Movie 2: **3D reconstruction of an acinus in S1 cells in 3D-**Matrigel drip **culture**

Beta-catenin is stained in red and nuclei are stained in blue. Green and yellow dots (some overlap with beta-catenin staining) corresponding to ZO-1 localization against the lumen are seen in the center of the multicellular structure when going through the optical sections. The lumen in this reconstruction appears as the black area in the center of the multicellular structure (it is the lumen modeled in the same acinus shown in movie 1).

Movie 3: **3D reconstruction of an acinus formed by S1 cells in HTP culture**

Moving through optical sections of staining for the cell nucleus (DNA in blue), cell-cell contact (beta-catenin in red) and apical region (ZO-1 in green) outlines the

concentration of ZO-1 around a central tiny lumen (that appears free of staining in a triangular shape). This particular lot of antibody against ZO-1 also shows some nuclear localization of ZO-1 in addition to the strong concentration at the apical side of the acinus (this staining pattern seemed to depend on the antibody lot and sometimes occurred also under 3D-Matrigel drip culture conditions). The apical concentration of ZO-1 appears yellow because it overlaps with the apical concentration of some of the beta-catenin staining.
